# Alternative translational initiation of ATP sulfurylase underlying dual localization of sulfate assimilation pathways in plastids and cytosol in *Arabidopsis thaliana*

**DOI:** 10.3389/fpls.2014.00750

**Published:** 2015-01-05

**Authors:** Anne-Sophie Bohrer, Naoko Yoshimoto, Ai Sekiguchi, Nicholas Rykulski, Kazuki Saito, Hideki Takahashi

**Affiliations:** ^1^Department of Biochemistry and Molecular Biology, Michigan State UniversityEast Lansing, MI, USA; ^2^Graduate School of Pharmaceutical Sciences, Chiba UniversityChiba, Japan; ^3^RIKEN Center for Sustainable Resource ScienceYokohama, Japan

**Keywords:** sulfur metabolism, ATP sulfurylase, alternative translational initiation, dual localization, *Arabidopsis*

## Abstract

Plants assimilate inorganic sulfate into sulfur-containing vital metabolites. ATP sulfurylase (ATPS) is the enzyme catalyzing the key entry step of the sulfate assimilation pathway in both plastids and cytosol in plants. *Arabidopsis thaliana* has four *ATPS* genes (*ATPS1, –2, –3*, and *–4*) encoding ATPS pre-proteins containing N-terminal transit peptide sequences for plastid targeting, however, the genetic identity of the cytosolic ATPS has remained unverified. Here we show that *Arabidopsis ATPS2* dually encodes plastidic and cytosolic ATPS isoforms, differentiating their subcellular localizations by initiating translation at AUG^Met1^ to produce plastid-targeted ATPS2 pre-proteins or at AUG^Met52^ or AUG^Met58^ within the transit peptide to have ATPS2 stay in cytosol. Translational initiation of ATPS2 at AUG^Met52^ or AUG^Met58^ was verified by expressing a tandem-fused synthetic gene, *ATPS2*_(5′*UTR-His12*)_*:Renilla luciferase:ATPS2*_(*Ile*13−*Val*77)_*:firefly luciferase*, under a single constitutively active CaMV 35S promoter in *Arabidopsis* protoplasts and examining the activities of two different luciferases translated in-frame with split N-terminal portions of ATPS2. Introducing missense mutations at AUG^Met52^ and AUG^Met58^ significantly reduced the firefly luciferase activity, while AUG^Met52^ was a relatively preferred site for the alternative translational initiation. The activity of luciferase fusion protein starting at AUG^Met52^ or AUG^Met58^ was not modulated by changes in sulfate conditions. The dual localizations of ATPS2 in plastids and cytosol were further evidenced by expression of ATPS2-GFP fusion proteins in *Arabidopsis* protoplasts and transgenic lines, while they were also under control of tissue-specific *ATPS2* promoter activity found predominantly in leaf epidermal cells, guard cells, vascular tissues and roots.

## Introduction

Sulfur is an essential macronutrient for plant growth, and it can be found in a wide variety of cellular components such as Cys, Met, GSH, sulfolipids, redox centers, and specialized metabolites involved in biotic and abiotic responses (Halkier and Gershenzon, 2006; Shimojima, 2011; Takahashi et al., 2011; Noctor et al., 2012). Sulfate is the main sulfur source available for plants in the environment. Therefore, reducing cofactors and carbon skeletons generated through photosynthesis are required in order to assimilate sulfate into organic sulfur metabolites. Following uptake of sulfate across the plasma membranes, several metabolic steps serve for reduction of sulfate. The first step of the sulfate assimilation pathway is catalyzed by ATP sulfurylase (ATPS) (ATP: sulfate adenylyltransferase, EC: 2.7.7.4) which uses ATP and sulfate to yield adenosine 5′-phosphosulfate (APS) and pyrophosphate. Following this step, APS is subsequently phosphorylated by APS kinase (APK, EC: 2.7.1.25) to form 3′-phosphoadenosine 5′-phosphosulfate (PAPS). PAPS is then used as a donor for sulfation reactions. Besides being utilized in the phosphorylation pathway, APS is supplied to the reductive assimilation pathway where it is converted to sulfite by APS reductase (APR, EC: 1.8.99.2). Sulfite is then reduced to sulfide by sulfite reductase (SiR, EC: 1.8.7.1). Cys biosynthesis from sulfide and *O*-acetylserine occurs following this reductive assimilation pathway.

ATPS is encoded by a multigenic family and its activity can be detected in cytosol and chloroplasts in plants (Lunn et al., 1990; Renosto et al., 1993; Klonus et al., 1994; Leustek et al., 1994; Murillo and Leustek, 1995; Logan et al., 1996; Hatzfeld et al., 2000; Rotte and Leustek, 2000; Phartiyal et al., 2006). APK is also found in both cytosol and plastids (Lee and Leustek, 1998; Lillig et al., 2001; Mugford et al., 2009). In contrast, reduction of APS catalyzed by APR occurs only in the plastids (Gutierrez-Marcos et al., 1996; Setya et al., 1996; Rotte and Leustek, 2000; Suter et al., 2000). APK and APR therefore compete for their common substrate APS in plastids, while no such competition happens in cytosol. PAPS biosynthesis in cytosol appears simple with ATPS and APK being direct enzymes involved in the pathway. However, it can eventually be affected by metabolic fluxes of APS phosphorylation and reduction in plastids, because PAPS is transported from plastids to cytosol (Gigolashvili et al., 2012). Thus, ATPS activities in plastids and cytosol contribute to provision of APS for downstream pathways in different ways, and their roles may vary depending on subcellular localizations.

Four *ATPS* genes (*ATPS1*, *–2*, *–3*, and *–4*) are present in the *Arabidopsis* genome (Leustek et al., 1994; Klonus et al., 1995; Murillo and Leustek, 1995; Logan et al., 1996; Hatzfeld et al., 2000). The protein coding regions of all four *ATPS* have the N-terminal leader sequences with characteristics for plastid-targeting transit peptides followed by the ATPS catalytic domains. Despite the presence of transit peptides in all four ATPS, the ATPS activity is detected in both chloroplasts and cytosol in *Arabidopsis* leaves (Rotte and Leustek, 2000). Thus, the identity of cytosolic ATPS has remained arguable, although *ATPS2* (Logan et al., 1996) has been proposed as a candidate gene to encode two isoforms (i.e., plastid- and cytosol-localizing ATPS) based on prediction of alternative translational initiation sites within the N-terminal transit peptide region (Hatzfeld et al., 2000).

In this study, we demonstrate experimental evidence that ATPS2 is alternatively translated into two different isoforms that dually localize in plastids and cytosol in *Arabidopsis*. The present study provides new insights into molecular mechanisms differentiating sulfate assimilation pathways in plastids and cytosol in plants.

## Materials and methods

### Chimeric gene constructs for protoplast transfection

Chimeric genes were generated using overlap-extension PCR methods. All the independent gene fragments were first amplified by PCRs using overlapping primers (Supplemental Table S1). The full-length chimeric genes were subsequently amplified by PCRs using 50 ng of each independent gene fragment, obtained from the initial PCRs, as templates and the primer pairs 1F/4R for *ATPS2-dual-Luc*, 1F/6R for *ATPS2_(5′UTR−Val77)_-GFP* and *ATPS2_FL_-GFP*, and 9F/6R for *ATPS1_(5′UTR−Val63)_-GFP* genes (Supplemental Table S1). All PCRs were performed using Platinum Pfx DNA Polymerase (Thermo Fisher Scientific). The resultant PCR-amplified chimeric genes were cloned into pCRBlunt II-TOPO vector (Thermo Fisher Scientific) and fully sequenced. Each *Bam*HI-*Not*I-ended chimeric gene was ligated with the *Bam*HI-*Not*I fragment of *p35S:GFP* vector using a ligation kit Ligation Mighty Mix (Takara Bio) to generate *p35S:ATPS2-dual-Luc*, *p35S:ATPS2_(5′UTR−Val77)_-GFP*, *p35S:ATPS2_FL_-GFP*, and *p35S:ATPS1_(5′UTR−Val63)_-GFP*.

The *p35S:GFP* vector [CaMV 35S:sGFP(S65T)] used in this study is a modified version of the *35Ω-sGFP(S65T)* vector (Chiu et al., 1996) from which the 35Ω promoter sequence was removed and replaced by the CaMV 35S promoter sequence of pBI221 (Clontech). The *Hind*III-*Bam*HI fragment (vector backbone, 3.65 kb) of *35Ω-sGFP(S65T)* and the *Hind*III-*Bam*HI fragment (CaMV 35S promoter, 0.8 kb) of pBI221 were ligated to obtain *p35S:GFP*.

Mutated versions of *p35S:ATPS2-dual-Luc* and *p35S:ATPS2_(5′UTR−Val77)_-GFP* were generated by site-directed mutagenesis using the QuickChange Lightning Site-Directed Mutagenesis Kit (Agilent) according to the manufacturer's instructions. Oligonucleotide primers and DNA templates that were used to introduce various point mutations are listed in Supplemental Tables S2, S3.

### Protoplast isolation and transfection

The protoplast isolation and transfections were performed as described by Yoo et al. (2007). *Arabidopsis* wild-type (Col-0) plants were grown on soil in an environment-controlled chamber under a 12-h-light / 12-h-dark cycle at 22°C with a light intensity of 80 μE m^−2^ s^−1^ and a relative humidity of 50%. For each protoplast transfection, 15–20 μg of plasmid DNAs were used, and the protoplasts were incubated in the transfection mixture for 10 min. Protoplasts were then re-suspended in WI solution (Yoo et al., 2007) supplemented with 1 mM MgSO_4_ (+S condition) or 1 mM MgCl_2_ (–S condition), and incubated in the dark for 16 h. Protoplasts were then harvested by centrifugation.

### Dual-luciferase assays

Dual luciferase assays were performed with the dual luciferase reporter assay system using the firefly luciferase reagent (LARII) and the *Renilla* luciferase reagent with firefly quenching (Stop & Glo) (Promega). All reagents were prepared as described by the manufacturer. Protoplasts were re-suspended in 50 μl 1X passive lysis buffer and incubated on ice for 15 min. The lysates were then centrifuged for 15 min at maximum speed at 4°C. Ten μl of undiluted supernatants were used to monitor the bioluminescence using a Centro SX3 luminometer (Berthold Technologies). Statistical significance was examined by One-Way analysis of the variance (ANOVA) and the Tukey's HSD *post-hoc* test with the level of significance set at 5%.

### Creation of transgenic plants expressing ATPS2 fused with GFP

For the creation of *ATPS2pro:ATPS2-GFP* fusion gene construct, oligonucleotide primers ATPS2-prom-FSal and ATPS2-CDSnstop-RNco (Supplemental Table S4) were used to amplify a genomic DNA fragment of *ATPS2* gene starting from 5′-region 3009-bp upstream of the plausible first translational initiation site and terminating just before the translational stop site. PCR was performed on genomic DNA prepared from *Arabidopsis thaliana* ecotype Col-0 using KOD plus DNA polymerase (Toyobo, Japan). The resultant PCR-amplified fragment of *ATPS2* was cloned into pCRBlunt II-TOPO (Thermo Fisher Scientific) and fully sequenced. The *Sal*I-*Nco*I-ended *ATPS2* gene fragment was inserted in the place of 35 Ω in the 35 Ω-sGFP(S65T) vector (Chiu et al., 1996) to obtain the *ATPS2pro:ATPS2-sGFP(S65T):NOSter* fusion gene. This fusion gene fragment was placed between the *Sal*I and *Eco*RI sites in the binary plasmid, pBI101 (Clontech), replacing the β-glucuronidase gene and the NOSter region (Figure S1).

The *ATPS2pro:ATPS2-GFP* chimeric gene constructs with mutated versions of *ATPS2* were created as follows. The *Bam*HI and *Xba*I sites, respectively located 748-bp upstream and 1127-bp downstream of the first translational initiation site of *ATPS2*, were used to cut out a DNA fragment from the binary plasmid harboring the *ATPS2pro:ATPS2-sGFP(S65T):NOSter* fusion gene (Figure S1), and this *Bam*HI-*Xba*I fragment was used as a template for overlap-extension PCRs. The nucleotide sequences of primer pairs used for the first PCRs are shown in Supplemental Table S4. The fragments obtained from the first PCR were mixed and amplified by PCR using primers ATPS2(–753)-FBam and ATPS2(+1136)-RXba. The DNA templates and the pairs of primers used for the construction of the mutated versions of *ATPS2pro:ATPS2-sGFP(S65T):NOSter* fusion gene are detailed in the Supplemental Table S5. The resultant PCR fragments containing mutations in *ATPS2* were cloned into pCR-Blunt II-TOPO and fully sequenced. The mutated *ATPS2* fragments were cut out as *Bam*HI-*Xba*I fragments, and used to replace the corresponding region of wild-type *ATPS2* gene in the binary plasmid harboring the *ATPS2pro:ATPS2-sGFP(S65T):NOSter* fusion gene construct (Figure S1).

The binary plasmids were transferred to *Agrobacterium tumefaciens* C58C1 GV3101 (pMP90) (Koncz and Schell, 1986) by a freeze-thaw method (Höfgen and Willmitzer, 1988). *Arabidopsis thaliana* ecotype Col-0 plants were transformed by a floral dip method (Clough and Bent, 1998). Transgenic plants were selected on GM agar medium (Valvekens et al., 1988) containing 50 mg L^−1^ kanamycin sulfate. Kanamycin-resistant T_2_ progenies were used for the analyses.

### Microscopy and imaging of GFP

Fluorescence of ATPS2-GFP fusion proteins in protoplasts and transgenic plants was observed using confocal laser-scanning microscopes, Fluoview FV10i (Olympus) and LSM510 (Zeiss).

### Protein extraction and immunoblot analysis

Total protein was prepared from leaves and roots of plants grown for 2 weeks on GM agar medium (Valvekens et al., 1988). Tissues were ground under liquid nitrogen and homogenized in the extraction buffer [50 mM Tris-MES (pH 7.5), 300 mM sucrose, 150 mM NaCl, 10 mM CH_3_COOK, 5 mM EDTA, 20 μM leupeptine, 100 μM 4-(2-aminoethyl)benzenesulfonyl fluoride, 1 mM phenylmethylsulfonyl fluoride]. The lysate was centrifuged at 10,000 g for 15 min, and the supernatant was collected. Protein concentrations were determined using a Bio-Rad protein assaying kit (Bio-Rad) based on the Bradford method (Bradford, 1976), using bovine serum albumin as a standard. Proteins were separated in 10% (w/v) polyacrylamide gel, and transferred to Immobilon-P membrane (Millipore) by electroblotting. Ten micrograms of crude proteins were loaded to each lane of the gels. The blot was incubated with anti-GFP mouse monoclonal antibody (Nacalai Tesque, Japan), followed by incubation with goat anti-mouse IgG conjugated to alkaline phosphatase (Promega). The presence of immuno-reactive protein was detected through the use of 5-bromo-4-chloro-3-indolyl-phosphate and nitro blue tetrazolium (Promega).

### Accession numbers

The reference sequence information on *Arabidopsis ATPS* gene family members is available at The Arabidopsis Information Resource (TAIR, http://www.arabidopsis.org/) under the following accession numbers: *ATPS1* (At3g22890); *ATPS2* (At1g19920); *ATPS3* (At4g14680); *ATPS4* (At5g43780).

## Results

### Translation of ATPS2 can be initiated from internal start sites

**Figure 1:**
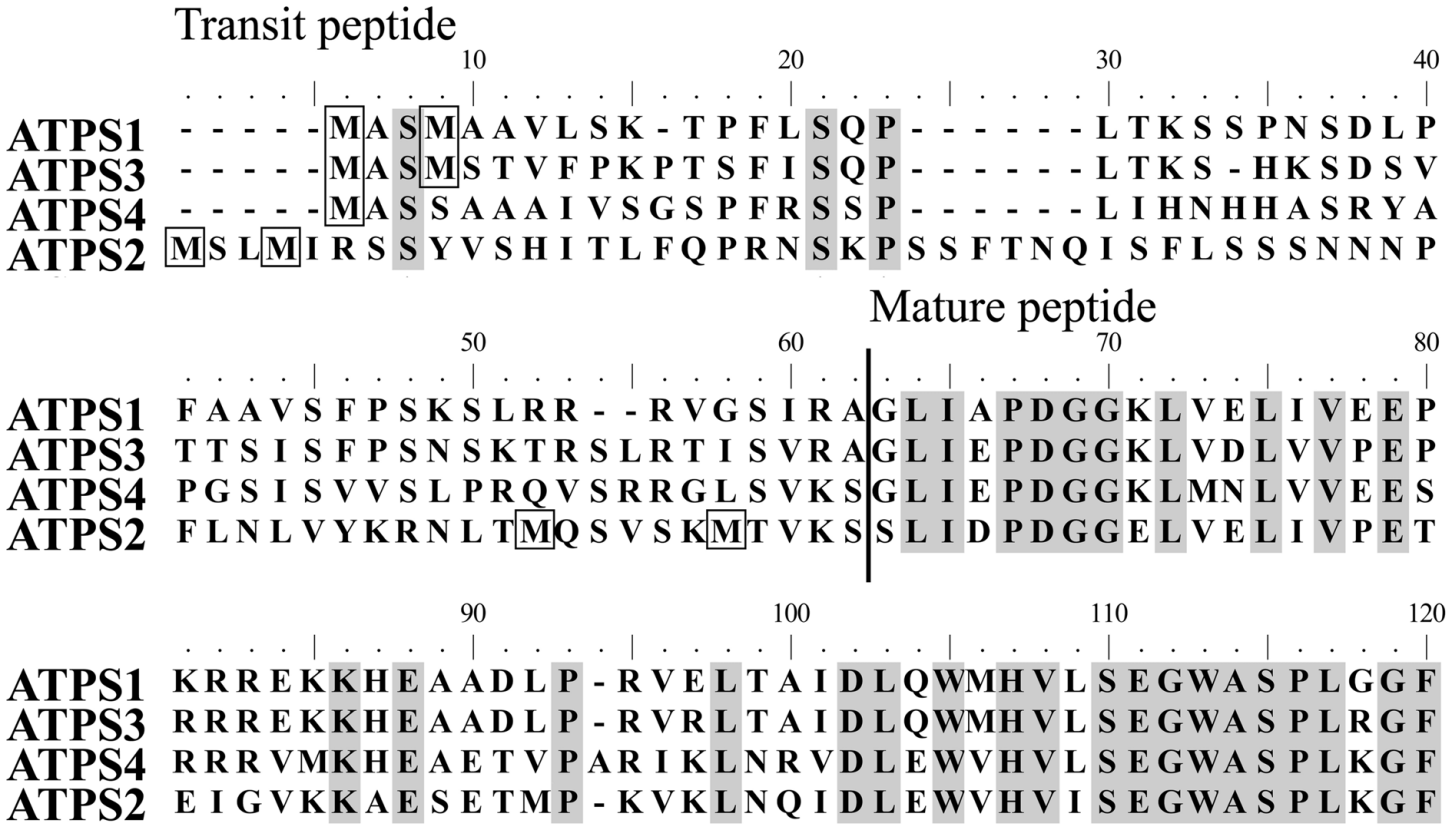
**Alignment of *Arabidopsis* ATP sulfurylase (ATPS) proteins**. *Arabidopsis thaliana* ATPS complete protein sequences were aligned using Clustal W2. The predicted cleavage site of the transit peptide for plastid targeting is indicated. Identical residues in all four sequences are shaded and methionine residues in the transit peptides are boxed.

The alignment of *Arabidopsis* ATPS protein sequences indicated that all four pre-proteins contained N-terminal extensions suggested to function as transit peptides for plastid targeting of polypeptides (Figure 1). Among them, only ATPS2 pre-protein contained four Met residues in its transit peptide, corresponding to Met1, Met4, Met52, and Met58 (Figure 1). The analysis of the nucleotide sequences surrounding AUG^Met1^, AUG^Met52^, and AUG^Met58^ codons indicated high similarities with the consensus sequence around translational initiation sites in dicot plants (Joshi et al., 1997; Hatzfeld et al., 2000). Translation of *ATPS2* mRNA was therefore predicted to start at multiple sites, AUG^Met1^ and either AUG^Met52^ or AUG^Met58^, to produce the plastidic and the cytosolic ATPS2 isoforms, respectively, in *Arabidopsis*.

**Figure 2:**
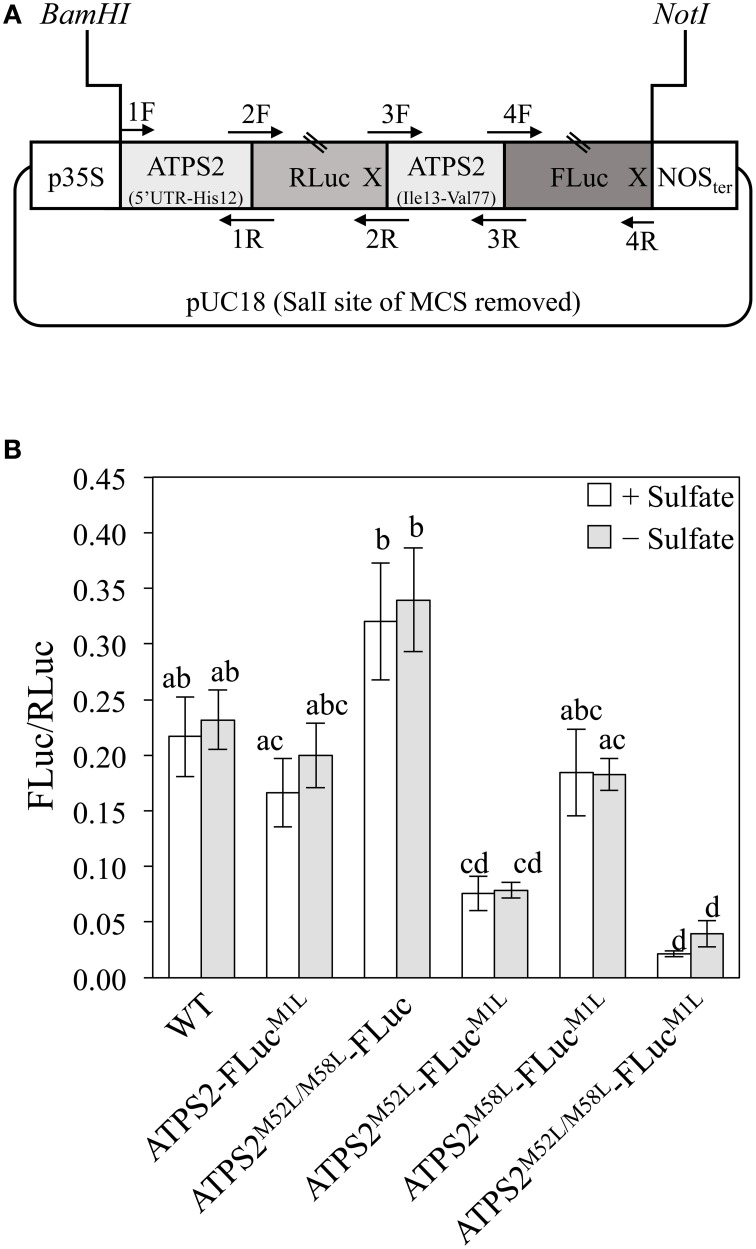
**Alternative translational initiation sites of ATPS2 determined by expression of dual-luciferase-tagged fusion constructs in *Arabidopsis* protoplasts. (A)** Schematic representation of *p35S:ATPS2-dual-Luc* used for expression of tandem fusion gene *ATPS2_(5′UTR-His12)_:Renilla luciferase:ATPS2_(Ile13−Val77)_:firefly luciferase*. Positions of annealing sites of primers (1F-4R) used for chimeric gene construction and stop codons (X) are indicated. **(B)** FLuc activity of protein extracts from *Arabidopsis* protoplasts transfected with either wild-type (WT) or mutated versions of *p35:ATPS2-dual-Luc* chimeric genes. Results are presented as FLuc/Rluc activity ratios in the same transfection. Values indicate mean ± SD for the results of 8 independent transfections. Values marked with different letters indicate statistically significant differences (ANOVA followed by Tukey's HSD *post-hoc* test; *p* < 0.05).

In order to test if the translation of ATPS2 can be alternatively initiated from either AUG^Met52^ or AUG^Met58^, we constructed a tandem fusion gene, *p35S:ATPS2-dual-Luc*, that splits the portion from the 5′-untranslated region (5′UTR) through the N-terminal 77-amino-acid region of ATPS2 into two parts fused separately to two luciferase reporters (Figure 2A). A 184-bp fragment of DNA, from the 5′UTR to His12 of ATPS2, was cloned in frame with the coding sequence of *Renilla* luciferase (RLuc), and the remaining 195-bp fragment, from Ile13 to Val77 of ATPS2, was cloned in frame with the coding sequence of firefly luciferase (FLuc). This fusion construct was designed to express a tandem-fused single mRNA from the CaMV 35S promoter, and to observe subsequent translation of that transcription unit into two different luciferase-fusion proteins. The fusion construct was transfected into *Arabidopsis* protoplasts and both luciferase activities were monitored to determine the presence of translational products, M1M4-RLuc and M52M58-FLuc. A similar bicistronic gene construct containing GFP and FLuc open reading frames has been used to characterize the ability of a viral internal ribosome entry site (IRES) to mediate cap-independent internal translational initiation in plants (Urwin et al., 2000).

The FLuc activity detected in the protoplasts indicated that M52M58-FLuc protein is produced (Figure 2B) and that an alternative translational initiation site is present and functional. To determine which of the potential start sites, AUG^Met52^ or AUG^Met58^, can be used for producing the cytosolic isoform of ATPS2 (cyt-ATPS2), missense point mutations were introduced to the AUG codons. When the AUG^Met1^ of FLuc was mutagenized to CUG^Leu^ (ATPS2-FLuc^M1L^), the FLuc/RLuc ratios were similar to or slightly lower than those in the wild type (WT). In contrast, when both AUG^Met52^ and AUG^Met58^ were mutagenized to CUG^Leu^ (ATPS2^M52L/M58L^-FLuc), the FLuc/RLuc ratios were higher than in the WT. These results suggest that the endogenous AUG^Met1^ of FLuc is preferentially used as a translation initiation site in this fusion construct. Therefore, AUG^Met1^ of FLuc was included in mutant constructs to make cross comparisons with or among the double and triple mutants. The comparisons among the three experimental groups, ATPS2-FLuc^M1L^, ATPS2^M52L^-FLuc^M1L^, and ATPS^M58L^-FLuc^M1L^, indicated that the mutation of AUG^Met52^ had the stronger impact showing approximately a 50% decrease in FLuc/RLuc ratios (Figure 2B). Moreover, the comparisons among ATPS2^M52L/M58L^-FLuc^M1L^, ATPS2^M52L^-FLuc^M1L^, and ATPS^M58L^-FLuc^M1L^ indicated that AUG^Met52^ was significant for translational initiation compared to AUG^Met58^ that only had a marginal effect (Figure 2B). These results indicated that translation of mRNA to FLuc fusion protein occurred more efficiently at AUG^Met52^ than AUG^Met58^. The results obtained from this experimental system suggest that *ATPS2* mRNA can be translated preferentially at AUG^Met52^ to form the cytosolic ATPS2 isoform, M52-ATPS2, rather than starting at AUG^Met58^ producing M58-ATPS2. In protoplasts expressing ATPS2^M52L/M58L^-FLuc^M1L^ fusion construct, FLuc activity equivalent to approximately 10% of the wild-type level was still detectable. Therefore, it cannot be ruled out that translation might have initiated at another AUG downstream of AUG^Met1^ to produce a functional FLuc protein, resulting in a low residual FLuc activity. In this experiment, the protoplasts were divided into two fractions following the transfection, and incubated under sulfate-sufficient (+Sulfate) or sulfate-deficient conditions (–Sulfate). However, the FLuc activity was not modulated by changes in sulfate conditions.

### ATPS2-GFP is dually localized in chloroplasts and cytosol

**Figure 3:**
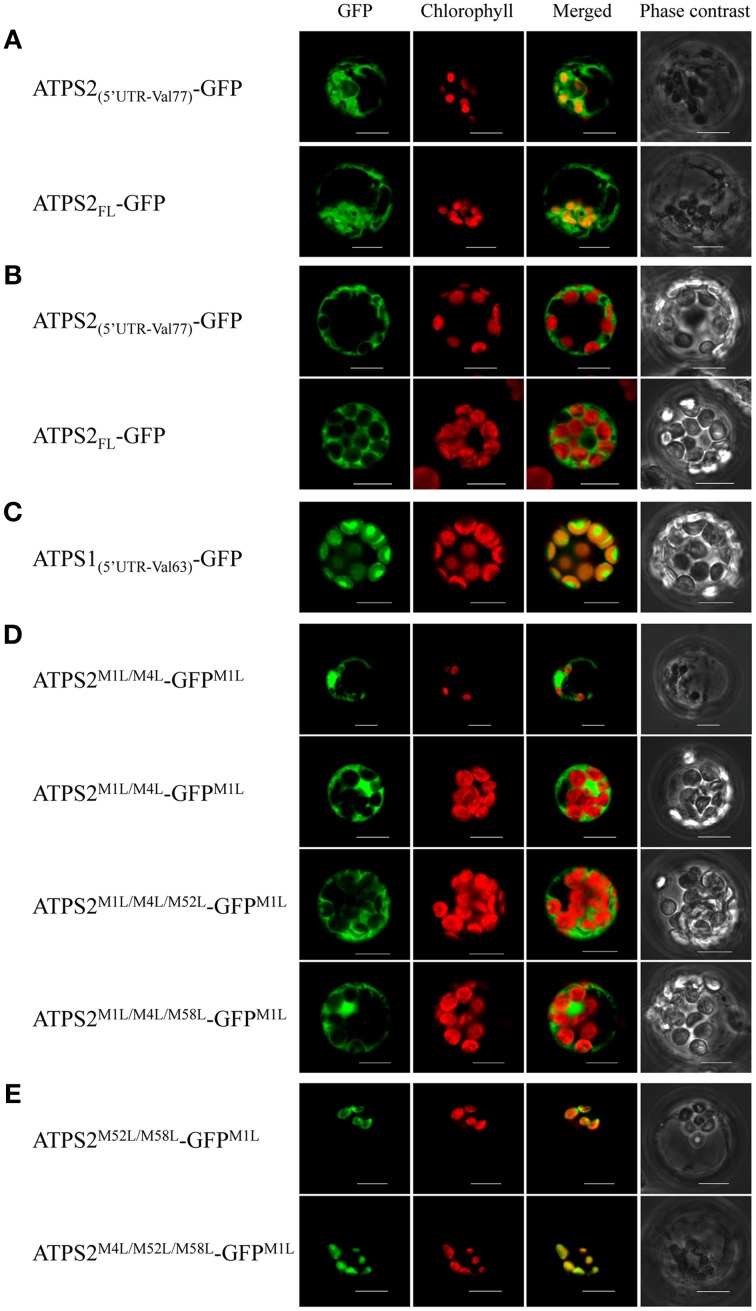
**Subcellular localization of ATPS2-GFP fusion proteins in *Arabidopsis* protoplasts. (A)** Chloroplast-cytosol dual-localization of ATPS2-GFP (ATPS2_(5′UTR-Val77)_-GFP and ATPS2_FL_-GFP). **(B)** Cytosolic localization of ATPS2-GFP (ATPS2_(5′UTR-Val77)_-GFP and ATPS2_FL_-GFP) in mesophyll protoplasts. **(C)** Chloroplastic localization of ATPS1-GFP (ATPS1_(5′UTR-Val63)_-GFP) in mesophyll protoplasts. **(D)** Cytosolic localization of ATPS2^M1L/M4L^-GFP^M1L^, ATPS2^M1L/M4L/M52L^-GFP^M1L^, and ATPS2^M1L/M4L/M58L^-GFP^M1L^. **(E)** Chloroplastic localization of ATPS2^M52L/M58L^-GFP^M1L^ and ATPS2^M4L/M52L/M58L^-GFP^M1L^. Fluorescence was detected using a confocal laser-scanning microscope. GFP fluorescence (green), chlorophyll fluorescence (red), merged images (green and red) and bright-field phase contrast images are shown. Scale bars = 10 μm.

To determine the subcellular localizations of the alternatively translated products of ATPS2 (M1-ATPS2 and M52-ATPS2 or M58-ATPS2), the 5′UTR and the N-terminal 77-amino-acid region of ATPS2 (ATPS2_(5′UTR−Val77)_) or the ATPS2 full-length (ATPS2_FL_) sequences were fused to GFP, and resultant *p35S:ATPS2*-*GFP* constructs were transiently expressed in *Arabidopsis* protoplasts (Figure 3). The ATPS1-GFP fusion construct (ATPS1_(5′UTR−Val63)_-GFP), containing the 5′UTR and the N-terminal 63-amino-acid region of ATPS1 fused to GFP following a Val residue conserved with Val77 of ATPS2, was prepared for comparison.

In protoplasts expressing either form of wild-type ATPS2-GFP fusion proteins (ATPS2_(5′UTR−Val77)_-GFP or ATPS2_FL_-GFP; Figures 3A), GFP signals were observed in both cytosol and chloroplasts (“GFP” column), where they were shown as GFP fluorescence excluded from or overlapping with chlorophyll fluorescence (“Merged” column). However, in the same transfection with the ATPS2-GFP fusion constructs, the GFP signals were found to be localized only in the cytosol in mesophyll protoplasts that contained a large number of fully developed chloroplasts (Figure 3B), unlike the dual localizations observed in protoplasts containing only a few small chloroplasts (Figure 3A). In contrast, the transfection of ATPS1_(5′UTR−Val63)_-GFP showed exclusive localization of GFP in the chloroplasts (Figure 3C).

To investigate the roles of potential start codons within the transit peptide region of ATPS2 in differentiating its localization to chloroplasts and/or cytosol, missense point mutations changing the AUG^Met^ start codons to CUG^Leu^ were introduced to ATPS2_(5′UTR−V77)_-GFP and these mutant forms were expressed in protoplasts. When CUG^Leu^ were introduced to both AUG^Met1^ and AUG^Met4^ in addition to AUG^Met1^ of GFP (ATPS2^M1L/M4L^-GFP^M1L^), GFP was exclusively localized in the cytosol of both cell-types with fully developed chloroplasts and small chloroplasts (Figure 3D). Furthermore, the protoplasts expressing the fusion constructs with either of these start codons intact (ATPS2^M1L/M4L/M52L^-GFP^M1L^ or ATPS2^M1L/M4L/M58L^-GFP^M1L^) showed GFP fluorescence localized only in the cytosol (Figure 3D). These results indicate that cytosolic ATPS2 isoform can be translated from either AUG^Met52^ or AUG^Met58^.

In contrast, the expression of a fusion protein ATPS2^M52L/M58L^-GFP^M1L^ in protoplasts indicated chloroplastic localization of GFP (Figure 3E). The GFP signals were found only in protoplasts containing a few small chloroplasts, similar to the results showing dual localizations driven by the native forms of ATPS2-GFP (Figure 3A). Subcellular localizations of GFP fusion proteins were further tested using mutants, ATPS2^M1L/M52L/M58L^-GFP^M1L^ and ATPS2^M4L/M52L/M58L^-GFP^M1L^. When AUG^Met4^ was mutated, GFP was still expressed and exclusively localized in these small chloroplasts (ATPS2^M4L/M52L/M58L^-GFP^M1L^; Figure 3E). However, when AUG^Met1^ was mutated, no GFP fluorescence could be detected (data not shown since no GFP signals were found as in non-transfected protoplasts). This indicates that the translation of chloroplastic ATPS2 pre-protein can only be initiated from the AUG^Met1^ start codon.

### Tissue and subcellular localizations of ATPS2-GFP in plants

**Figure 4:**
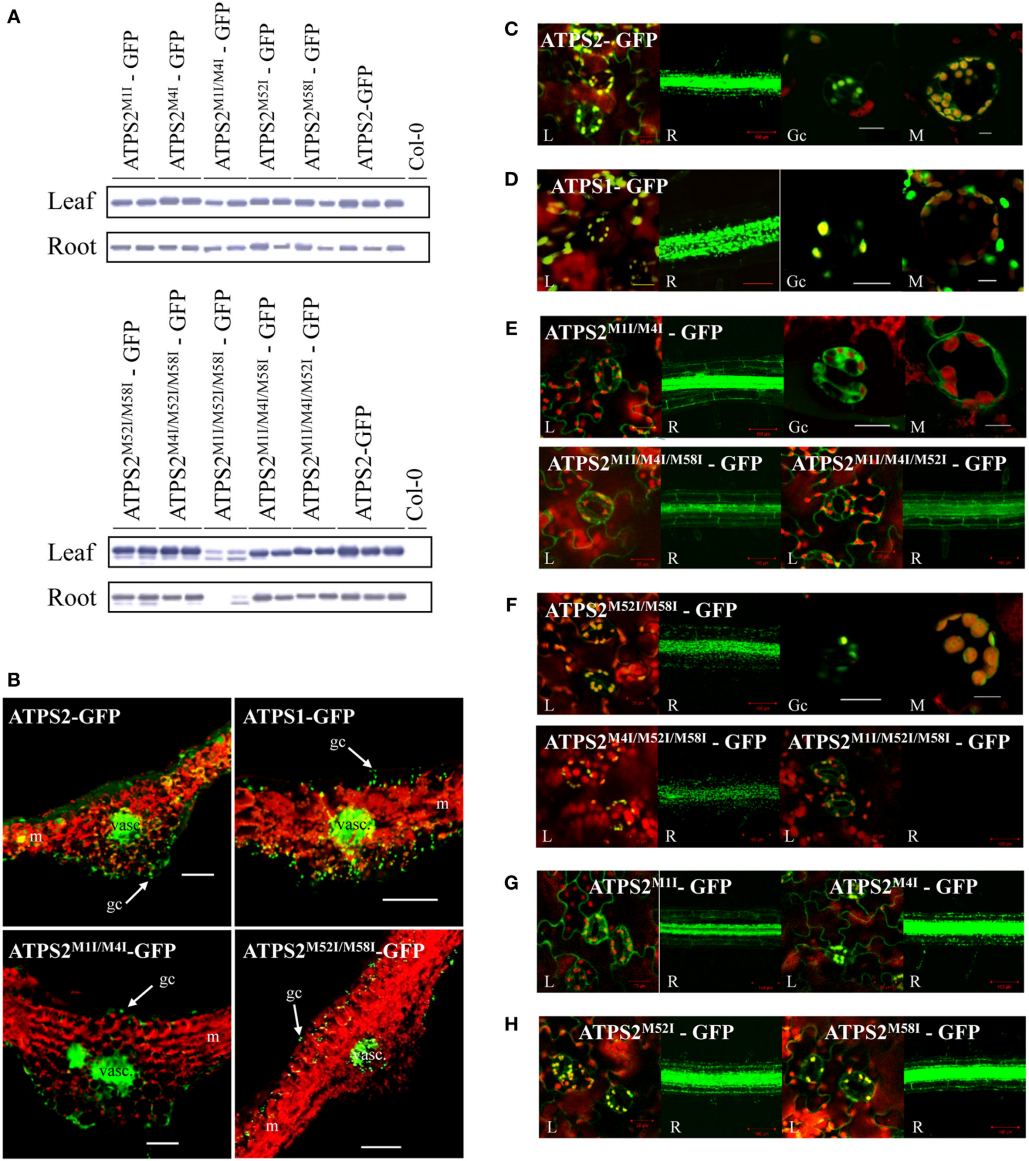
**Tissue and subcellular localization of ATPS2-GFP fusion proteins in *Arabidopsis* transgenic plants. (A)** Detection of ATPS2-GFP fusion proteins in each transgenic line by western blotting using anti-GFP antibody. **(B)** GFP fluorescence (green) observed in transverse sections of leaves from the transgenic lines expressing *ATPS1pro:ATPS1:GFP*, *ATPS2pro:ATPS2:GFP* and mutated versions (ATPS2^M1I/M4I^-GFP and ATPS2^M52I/M58I^-GFP). Red indicates chlorophyll autofluorescence. Guard cells (gc), mesophyll cells (m) and vascular tissues (vasc) are indicated. Scale bars = 100 μm. **(C–H)** Subcellular localizations of GFP fluorescence (green) in leaves (L), roots (R), guard cells (Gc), and mesophyll cells (M). Red indicates chlorophyll autofluorescence. Yellow or orange indicates overlap between green and red signals. Scales bars = 20 μm (for leaves), 100 μm (for roots), and 10 μm (for guard cells and mesophyll cells).

To further study the spatial and subcellular localization of ATPS2 in plants, *ATPS2pro:ATPS2-GFP* fusion constructs with or without the mutations of translational start sites were prepared (Figure S1), and stable *Arabidopsis* transgenic lines were obtained for the microscopic analysis (Figure 4). The transgenic lines expressing the *ATPS1pro:ATPS1-GFP* fusion construct (Kawashima et al., 2011) were used for comparison. These chimeric constructs are designed to express full-length ATPS-GFP fusion proteins under control of native *ATPS* promoters in *Arabidopsis*.

The accumulations of ATPS2-GFP fusion proteins in transgenic lines were monitored by western blotting using anti-GFP antibody (Figure 4A). The fusion proteins were detected in leaves and roots of all lines except ATPS2^M1I/M52I/M58I^-GFP, in which the fusion proteins were barely detectable in roots and only slightly produced in leaves (Figure 4A), despite the mRNAs being almost equally accumulated as those in transgenic lines made with other fusion constructs (Figure S2).

In transgenic lines expressing ATPS2-GFP fusion proteins, strong GFP fluorescence was observed in epidermal cells and guard cells as well as in vascular tissues and parenchyma cells on their abaxial side (Figure 4B). The same patterns of expression were observed in plants expressing the mutant constructs, ATPS2^M1I/M4I^-GFP or ATPS2^M52I/M58I^-GFP, prepared to determine their cytosolic or chloroplastic localizations of GFP fusion proteins (Figure 4B). The transgenic lines expressing the *ATPS1pro:ATPS1-GFP* fusion construct (Kawashima et al., 2011) also showed similar tissue localization patterns of GFP (Figure 4B). In all transgenic lines, the signals of GFP were much weaker in mesophyll cells, although they were detectable.

At subcellular levels, signals of GFP were dually localized in plastids and cytosol in both leaves and roots of ATPS2-GFP lines (Figure 4C), while they were present only in plastids in ATPS1-GFP lines (Figure 4D). When missense mutations (AUC^Ile^) were introduced to both AUG^Met1^ and AUG^Met4^ (ATPS2^M1I/M4I^-GFP), GFP was localized exclusively in the cytosol in both leaves and roots (Figure 4E). Moreover, in transgenic lines expressing ATPS2^M1I/M4I/M58I^-GFP or ATPS2^M1I/M4I/M52I^-GFP fusion proteins, the GFP fluorescence was similarly detected only in the cytosol (Figure 4E). In contrast, in transgenic lines expressing the mutant construct, ATPS2^M52I/M58I^-GFP, the fluorescence of GFP was localized exclusively in chloroplasts (Figure 4F). The same pattern of GFP localization was observed with ATPS2^M4I/M52I/M58I^-GFP (Figure 4F). However, in ATPS2^M1I/M52I/M58I^-GFP transgenic lines, GFP fluorescence could not be detected in roots, and the GFP signals in the chloroplasts in leaves were very weak (Figure 4F). The results were consistent with the low levels of ATPS2^M1I/M52I/M58I^-GFP fusion protein accumulation shown in the western blots (Figure 4A). The significance of AUG^Met1^ for the translational initiation of chloroplast-targeted isoform was also indicated by localization of GFP signals found only in the cytosol in leaves and roots of ATPS2^M1I^-GFP lines (Figure 4G). In contrast, the point mutation at AUG^Met4^ resulted in dual localization maintained as in the wild-type ATPS2-GFP, suggesting that AUG^Met1^, AUG^Met52^, and AUG^Met58^ are viable as start codons (Figure 4G). The single point mutations at AUG^Met52^ or AUG^Met58^ showed similar dual localizations (Figure 4H), supporting the results obtained from triple mutants showing functional redundancies of these two start sites to produce the cytosolic isoform (Figure 4E).

## Discussion

The sequence analysis of four ATPS pre-proteins of *Arabidopsis* points to a uniqueness of ATPS2 containing four in-frame AUG codons within its transit peptide region (Met1, Met4, Met52, and Met58) (Figure 1). The present study demonstrates that translation of *ATPS2* mRNA starts at multiple AUG^Met^ translational initiation sites and produces plastidic and cytosolic ATPS2 isoforms in *Arabidopsis* (Figures 2–4). This appears to happen when the nucleotide contexts surrounding the AUG start codons are favorable for initiating translations. Several studies report that alternative translations of a single mRNA can produce protein isoforms located in different subcellular compartments in plants. Polγ2 organellar DNA polymerases are localized in chloroplast and mitochondria following alternative translational initiation (Wamboldt et al., 2009). AtMBP-1 is alternatively translated from *LOS2* transcript at the internal start codon and localizes to nucleus to modulate expression of transcriptional repressor for cold acclimation, whereas the full-length protein LOS2 functions as enolase in the glycolytic pathway in the cytosol (Lee et al., 2002; Kang et al., 2013). With regard to metabolic enzymes, NAD(P)HX dehydratase and epimerase are shown to localize in mitochondria, plastids, and cytosol by using alternative translational initiation sites (Niehaus et al., 2014).

Multiple lines of experimental evidence indicate alternative translation of ATPS2 and its relevance to plastid-cytosol dual subcellular localizations. Expression of a tandem luciferase fusion gene *ATPS2_(5′UTR−His12)_:Renilla luciferase:ATPS2_(Ile13−Val77)_:firefly luciferase* in *Arabidopsis* protoplasts suggests that M52M58-FLuc fusion protein is produced by translation initiated at internal start sites, AUG^Met52^ or AUG^Met58^ (Figure 2). Point mutations of these alternative start sites further suggest AUG^Met52^ being the preferred site over AUG^Met58^. Furthermore, subcellular localizations of ATPS2-GFP proteins show alternative translational initiation underlying plastid-cytosol dual localizations (Figures 3, 4). The point mutation of AUG^Met1^ suggests the presence of this start site being essential for initiating the translation of ATPS2 pre-protein targeted to plastids. In contrast, both AUG^Met52^ and AUG^Met58^ are capable of initiating the translation of cytosolic ATPS2 isoform. These results clearly suggest *bona fide* relationships between the translational start sites and the duality of subcellular localizations of ATPS2 in *Arabidopsis*.

The exact mechanism that explains the alternative translation of *ATPS2* mRNA yet remains to be verified. Leaky ribosome scanning may be one of the possible scenarios where the same ribosome reads through the entire *ATPS2* mRNA to generate two ATPS2 protein isoforms with distinct subcellular localizations. Wamboldt et al. (2009) describes that such a mechanism of alternative translation of organellar DNA polymerase *Polγ2* mRNA allows dual localization of its translated products to mitochondria and chloroplasts in *Arabidopsis*. The other possible mechanism would be the presence of an internal ribosome entry site (IRES) around AUG^Met52^ and AUG^Met58^, allowing *ATPS2* mRNA to produce the cytosolic ATPS2 independent of the plastid-targeted ATPS2 pre-protein. It is suggested that an IRES element in the 5′UTR mediates cap-independent selective translation of a maize heat shock protein *Hsp101* mRNA during heat stress (Dinkova et al., 2005). In addition to these mechanisms, translation efficiency of a bicistronic mRNA may be affected by alteration of sequence contexts having RLuc between the first (AUG^Met1^) and alternative (AUG^Met52^ and AUG^Met58^) start codons (Figure 2A). The overall stability of a long bicistronic mRNA with an internal stop codon can also be reduced by nonsense-mediated decay. Molecular mechanisms of alternative translational initiations need to be investigated with precautions of considering these additional possibilities.

The transient expression of *p35S:ATPS2*-*GFP* fusion gene in *Arabidopsis* protoplasts indicates two patterns of subcellular localizations of GFP fluorescence depending on cell types: the dual localization is observed in protoplasts that contained a limited number of small chloroplasts, most likely derived from tissues that are less active in photosynthesis (Figure 3A), whereas the signal of GFP is found only in the cytosol of protoplasts containing a large number of fully developed chloroplasts, i.e., mesophyll cell protoplasts (Figure 3B). It is notable that such differential patterns of subcellular localizations are not seen with ATPS1-GFP (Figure 3C). The *Arabidopsis* transgenic lines expressing *ATPS2pro:ATPS2-GFP* fusion gene further demonstrate plastid-cytosol dual localization of ATPS2-GFP *in planta*. Mutations of AUG^Met1^, AUG^Met52^, and/or AUG^Met58^ unequivocally indicate requirement of these potential translational start sites for producing the isoforms localized to plastids and cytosol. The analysis of transgenic lines reveal that ATPS2-GFP is dually localized with its expression being the highest in epidermal cells, guard cells, vascular tissues including bundle sheath cells, and parenchyma cells present on the abaxial side of vasculature, which are considered less active in photosynthesis (Figure 4B). This pattern of expression is partly consistent with transcriptome data indicating relatively higher expression of *ATPS2* in guard cells than in mesophyll cells (Arabidopsis eFP Browser, http://bar.utoronto.ca/efp/cgi-bin/efpWeb.cgi). These results are likely consistent with dual subcellular localizations observed in protoplasts with small chloroplasts (Figure 3A). ATPS2-GFP is still found dually localized in mesophyll cells of transgenic lines (Figure 4C), although the level of expression is lower than those observed in the cell-types or tissues described above (Figure 4B). The subcellular localization of ATPS2-GFP in mesophyll protoplasts, which appears exclusive to the cytosol (Figure 3B), must therefore be speculated to have happened under unique mechanisms. Ectopically expressed *ATPS2-GFP* could have been selectively translated to the cytosolic isoform or post-translationally regulated to localize the polypeptides in cytosol but not in chloroplasts under specific conditions in protoplasts. Such mechanisms that may partially differentiate subcellular localizations in different cells appear specific to ATPS2, since our results indicate that transient expression of *p35S-ATPS1-GFP* in mesophyll protoplasts demonstrates clear localization of GFP signals in chloroplasts (Figure 3C) nevertheless cell-type specificities of gene expressions are similar between *ATPS1* and *ATPS2* in stable transformants (Figure 4B).

As mentioned above, both ATPS1 and ATPS2 are mainly expressed in epidermal cells, guard cells, vascular tissues, and cells in their vicinity (i.e., parenchyma cells on the abaxial side of the vasculature), but to a lower extent in mesophyll cells (Figure 4B). These patterns of expression of ATPS are similar to those of APK in leaves and roots of *Arabidopsis* (Mugford et al., 2009). Spatial co-localization of ATPS and APK is likely in accordance with their roles in providing PAPS for secondary metabolism producing sulfated compounds. It is known that, upon herbivore attack, one of the major sulfated compounds produced in Brassicaceae are glucosinolates (GLs). GLs are hydrolyzed by myrosinase and the by-products generated upon their hydrolysis serve as defense molecules against herbivores. Although GLs are found in the entire leaf, their abundance is higher in tissues surrounding mid-veins and in the periphery of leaf (Shroff et al., 2008). The specificity of GLs distribution seems likely a mechanism to limit herbivore feeding, as they tend to feed from the edges of plant leaves. Moreover, myrosinase is localized in myrosin cells in the phloem parenchyma (Andréasson et al., 2001). The spatial separation of the myrosinase-GLs system prevents the unnecessary hydrolysis of GLs. The co-localization of ATPS and APK in the vasculature or in the vicinity of the cells expressing myrosinase is therefore indicative of its potential contribution to providing substrates for GLs biosynthesis. Furthermore, a recent study of the translatome of vascular bundle sheath cells highlights the key role of these cells in sulfur metabolism in *Arabidopsis* (Aubry et al., 2014).

With regard to the control of sulfate assimilation, ATPS and APK in plastids appear to be expressed in favor of synthesizing PAPS for GLs biosynthesis under sulfur-sufficient conditions. MiR395s involved in post-transcriptional gene silencing of the chloroplastic ATPS1, –3, and –4, are repressed under sulfur-sufficient conditions (Kawashima et al., 2009). Chloroplastic APK (APK1 and APK2) play significant roles in providing PAPS for GLs biosynthesis (Mugford et al., 2009) while APR is repressed under sulfur-sufficient conditions. In contrast, the physiological relevance of the presence of cytosolic ATPS and APK and their molecular regulatory mechanisms are not well-documented to date. The present study unravels the molecular identity of the cytosolic ATPS2 and proposes alternative translational initiation as an underlying mechanism for its emergence. This translational mechanism specifically allows plastid-cytosol dual localization of ATPS2, a unique non-miR395 target among the *ATPS* gene family members. It is noteworthy that such distinction of molecular control mechanisms is apparent among the *ATPS* family members nevertheless the cell-type specificity resembles each other. The results shown in this study suggest that alternative translational initiation of ATPS2 is not significantly modulated by changes in sulfur conditions. Control of chloroplastic ATPS (ATPS1, –3, and –4) thus seems important for regulation of PAPS biosynthesis in response to sulfate supply, although the potential of the miR395-mediated post-transcriptional regulation may be limited for fine-tuning the ATPS1, –3, and −4 transcript levels (Kawashima et al., 2011). The physiological role of the cytosolic ATPS2 remains to be elucidated with relevance to its function in balancing PAPS biosynthesis between plastids and cytosol.

### Conflict of interest statement

The authors declare that the research was conducted in the absence of any commercial or financial relationships that could be construed as a potential conflict of interest.

## References

[B1] Andréasson E. Bolt Jørgensen L. Höglund A.-S. Rask L. Meijer J. (2001). Different myrosinase and idioblast distribution in Arabidopsis and *Brassica napus*. Plant Physiol. 127, 1750–1763. 10.1104/pp.010334 11743118 PMC133578

[B2] Aubry S. Smith-Unna R. D. Boursnell C. M. Kopriva S. Hibberd J. M. (2014). Transcript residency on ribosomes reveals a key role for the *Arabidopsis thaliana* bundle sheath in sulfur and glucosinolate metabolism. Plant J. 78, 659–673. 10.1111/tpj.12502 24617819

[B3] Bradford M. M. (1976). A rapid and sensitive method for the quantitation of microgram quantities of protein utilizing the principle of protein-dye binding. Anal. Biochem. 72, 248–254. 10.1016/0003-2697(76)90527-3 942051

[B4] Chiu W. Niwa Y. Zeng W. Hirano T. Kobayashi H. Sheen J. (1996). Engineered GFP as a vital reporter in plants. Curr. Biol. 6, 325–330. 10.1016/S0960-9822(02)00483-9 8805250

[B5] Clough S. J. Bent A. F. (1998). Floral dip: a simplified method for *Agrobacterium*-mediated transformation of *Arabidopsis thaliana*. Plant J. 16, 735–743. 10.1046/j.1365-313x.1998.00343.x 10069079

[B6] Dinkova T. D. Zepeda H. Martínez-Salas E. Martínez L. M. Nieto-Sotelo J. de Jiménez E. S. (2005). Cap-independent translation of maize Hsp101. Plant J. 41, 722–731. 10.1111/j.1365-313X.2005.02333.x 15703059

[B7] Gigolashvili T. Geier M. Ashykhmina N. Frerigmann H. Wulfert S. Krueger S.. (2012). The *Arabidopsis* thylakoid ADP/ATP carrier TAAC has an additional role in supplying plastidic phosphoadenosine 5′-phosphosulfate to the cytosol. Plant Cell 24, 4187–4204. 10.1105/tpc.112.101964 23085732 PMC3517245

[B8] Gutierrez-Marcos J. F. Roberts M. A Campbell E. I. Wray J. L. (1996). Three members of a novel small gene-family from *Arabidopsis thaliana* able to complement functionally an *Escherichia coli* mutant defective in PAPS reductase activity encode proteins with a thioredoxin-like domain and “APS reductase” activity. Proc. Natl. Acad. Sci. U.S.A. 93, 13377–13382. 10.1073/pnas.93.23.13377 8917599 PMC24101

[B9] Halkier B. A. Gershenzon J. (2006). Biology and biochemistry of glucosinolates. Annu. Rev. Plant Biol. 57, 303–333. 10.1146/annurev.arplant.57.032905.105228 16669764

[B10] Hatzfeld Y. Lee S. Lee M. Leustek T. Saito K. (2000). Functional characterization of a gene encoding a fourth ATP sulfurylase isoform from *Arabidopsis thaliana*. Gene 248, 51–58. 10.1016/S0378-1119(00)00132-3 10806350

[B11] Höfgen R. Willmitzer L. (1988). Storage of competent cells for *Agrobacterium* transformation. Nucleic Acids Res. 16:9877. 10.1093/nar/16.20.9877 3186459 PMC338805

[B12] Joshi C. P. Zhou H. Huang X. Chiang V. L. (1997). Context sequences of translation initiation codon in plants. Plant Mol. Biol. 35, 993–1001. 10.1023/A:1005816823636 9426620

[B13] Kang M. Abdelmageed H. Lee S. Reichert A. Mysore K. S. Allen R. D. (2013). AtMBP-1, an alternative translation product of LOS2, affects abscisic acid responses and is modulated by the E3 ubiquitin ligase AtSAP5. Plant J. 76, 481–493. 10.1111/tpj.12312 23952686

[B14] Kawashima C. G. Matthewman C. A. Huang S. Lee B.-R. Yoshimoto N. Koprivova A.. (2011). Interplay of SLIM1 and miR395 in the regulation of sulfate assimilation in Arabidopsis. Plant J. 66, 863–876. 10.1111/j.1365-313X.2011.04547.x 21401744

[B15] Kawashima C. G. Yoshimoto N. Maruyama-Nakashita A. Tsuchiya Y. N. Saito K. Takahashi H.. (2009). Sulphur starvation induces the expression of microRNA-395 and one of its target genes but in different cell types. Plant J. 57, 313–321. 10.1111/j.1365-313X.2008.03690.x 18801012

[B16] Klonus D. Höfgen R. Willmitzer L. Riesmeier J. W. (1994). Isolation and characterization of two cDNA clones encoding ATP-sulfurylases from potato by complementation of a yeast mutant. Plant J. 6, 105–112. 10.1046/j.1365-313X.1994.6010105.x 7920699

[B17] Klonus D. Riesmeier J. W. Willmitzer L. (1995). A cDNA clone for an ATP-sulfurylase from *Arabidopsis thaliana*. Plant Physiol. 107, 653–654. 10.1104/pp.107.2.653 7724678 PMC157172

[B18] Koncz C. Schell J. (1986). The promoter of T_*L*_-DNA gene 5 controls the tissue-specific expression of chimaeric genes carried by a novel type of *Agrobacterium* binary vector. Mol. Genet. Genomics 204, 383–396 10.1007/BF00331014

[B19] Lee H. Guo Y. Ohta M. Xiong L. Stevenson B. Zhu J.-K. (2002). *LOS2*, a genetic locus required for cold-responsive gene transcription encodes a bi-functional enolase. EMBO J. 21, 2692–2702. 10.1093/emboj/21.11.2692 12032082 PMC126021

[B20] Lee S. Leustek T. (1998). APS kinase from *Arabidopsis thaliana*: genomic organization, expression, and kinetic analysis of the recombinant enzyme. Biochem. Biophys. Res. Commun. 247, 171–175. 10.1006/bbrc.1998.8751 9636674

[B21] Leustek T. Murillo M. Cervantes M. Biology A. M. (1994). Cloning of a cDNA encoding ATP sulfurylase from *Arabidopsis thaliana* by functional expression in *Saccharomyces cerevisiae*. Plant Physiol. 105, 897–902. 10.1104/pp.105.3.897 8058839 PMC160738

[B22] Lillig C. H. Schiffmann S. Berndt C. Berken A. Tischka R. Schwenn J. D. (2001). Molecular and catalytic properties of *Arabidopsis thaliana* adenylyl sulfate (APS)-kinase. Arch. Biochem. Biophys. 392, 303–310. 10.1006/abbi.2001.2453 11488606

[B23] Logan H. M. Cathala N. Grignon C. Davidian J-C. (1996). Cloning of a cDNA encoded by a member of the *Arabidopsis thaliana* ATP Sulfurylase multigene family: expression studies in yeast and in relation to plant sulfur nutrition. J. Biol. Chem. 271, 12227–12233. 10.1074/jbc.271.21.12227 8647819

[B24] Lunn J. E. Droux M. Martin J. Douce R. (1990). Localization of ATP sulfurylase and *O*-acetylserine(thiol)lyase in spinach leaves. Plant Physiol. 94, 1345–1352. 10.1104/pp.94.3.1345 16667839 PMC1077384

[B25] Mugford S. G. Yoshimoto N. Reichelt M. Wirtz M. Hill L. Mugford S. T.. (2009). Disruption of adenosine-5′-phosphosulfate kinase in *Arabidopsis* reduces levels of sulfated secondary metabolites. Plant Cell 21, 910–927. 10.1105/tpc.109.065581 19304933 PMC2671714

[B26] Murillo M. Leustek T. (1995). Adenosine-5′-triphosphate-sulfurylase from *Arabidopsis thaliana* and *Escherichia coli* are functionally equivalent but structurally and kinetically divergent: nucleotide sequence of two adenosine-5′-triphosphate-sulfurylase cDNAs from *Arabidopsis thaliana* and anaysis of a recombinant enzyme. Arch. Biochem. Biophys. 323, 195–204. 10.1006/abbi.1995.0026 7487067

[B27] Niehaus T. D. Richardson L. G. L. Gidda S. K. ElBadawi-Sidhu M. Meissen J. K. Mullen R. T.. (2014). Plants utilize a highly conserved system for repair of NADH and NADPH hydrates. Plant Physiol. 165, 52–61. 10.1104/pp.114.236539 24599492 PMC4012604

[B28] Noctor G. Mhamdi A. Chaouch S. Han Y. Neukermans J. Marquez-Garcia B.. (2012). Glutathione in plants: an integrated overview. Plant Cell Environ. 35, 454–484. 10.1111/j.1365-3040.2011.02400.x 21777251

[B29] Phartiyal P. Kim W.-S. Cahoon R. E. Jez J. M. Krishnan H. B. (2006). Soybean ATP sulfurylase, a homodimeric enzyme involved in sulfur assimilation, is abundantly expressed in roots and induced by cold treatment. Arch. Biochem. Biophys. 450, 20–29. 10.1016/j.abb.2006.03.033 16684499

[B30] Renosto F. Patel H. C. Martin R. L. Christopher T. Zimmerman G. Segel I. H. (1993). ATP sulfurylase from higher plants: kinetic and structural characterization of the chloroplast and cytosol enzymes from spinach leaf. Arch. Biochem. Biophys. 307, 272–285. 10.1006/abbi.1993.1590 8274013

[B31] Rotte C. Leustek T. (2000). Differential subcellular localization and expression of ATP sulfurylase and 5′-adenylylsulfate reductase during ontogenesis of Arabidopsis leaves indicates that cytosolic and plastid forms of ATP sulfurylase may have specialized functions. Plant Physiol. 124, 715–724. 10.1104/pp.124.2.715 11027720 PMC59176

[B32] Setya A. Murillo M. Leustek T. (1996). Sulfate reduction in higher plants: molecular evidence for a novel 5′-adenylylsulfate reductase. Proc. Natl. Acad. Sci. U.S.A. 93, 13383–13388. 10.1073/pnas.93.23.13383 8917600 PMC24102

[B33] Shimojima M. (2011). Biosynthesis and functions of the plant sulfolipid. Prog. Lipid Res. 50, 234–239. 10.1016/j.plipres.2011.02.003 21371504

[B34] Shroff R. Vergara F. Muck A. Svatos A. Gershenzon J. (2008). Nonuniform distribution of glucosinolates in *Arabidopsis thaliana* leaves has important consequences for plant defense. Proc. Natl. Acad. Sci. U.S.A. 105, 6196–6201. 10.1073/pnas.0711730105 18408160 PMC2329684

[B35] Suter M. von Ballmoos P. Kopriva S. den Camp R. O. Schaller J. Kuhlemeier C.. (2000). Adenosine 5′-phosphosulfate sulfotransferase and adenosine 5′-phosphosulfate reductase are identical enzymes. J. Biol. Chem. 275, 930–936. 10.1074/jbc.275.2.930 10625629

[B36] Takahashi H. Kopriva S. Giordano M. Saito K. Hell R. (2011). Sulfur assimilation in photosynthetic organisms: molecular functions and regulations of transporters and assimilatory enzymes. Annu. Rev. Plant Biol. 62, 157–184. 10.1146/annurev-arplant-042110-103921 21370978

[B37] Urwin P. Yi L. Martin H. Atkinson H. Gilmartin P. M. (2000). Functional characterization of the EMCV IRES in plants. Plant J. 24, 583–589. 10.1046/j.1365-313x.2000.00904.x 11123797

[B38] Valvekens D. Van Montagu M. Van Lijsebettens M. (1988). *Agrobacterium tumefaciens*-mediated transformation of *Arabidopsis thaliana* root explants by using kanamycin selection. Proc. Natl. Acad. Sci. U.S.A. 85, 5536–5540. 10.1073/pnas.85.15.5536 16593964 PMC281793

[B39] Wamboldt Y. Mohammed S. Elowsky C. Wittgren C. de Paula W. B. M. Mackenzie S. A. (2009). Participation of leaky ribosome scanning in protein dual targeting by alternative translation initiation in higher plants. Plant Cell 21, 157–167. 10.1105/tpc.108.063644 19182105 PMC2648075

[B40] Yoo S.-D. Cho Y.-H. Sheen J. (2007). *Arabidopsis* mesophyll protoplasts: a versatile cell system for transient gene expression analysis. Nat. Protoc. 2, 1565–1572. 10.1038/nprot.2007.199 17585298

