# Supplementary material for: Alternative translational initiation of ATP sulfurylase underlying dual localization of sulfate assimilation pathways in plastids and cytosol in Arabidopsis thaliana
Source: Front Plant Sci. 2015 Jan 5;5:750. doi: 10.3389/fpls.2014.00750 (PMC4283515; doi:10.3389/fpls.2014.00750)
Supplement: Supplementary file 6 [file Presentation1.PDF]

Supplemental Figure S1

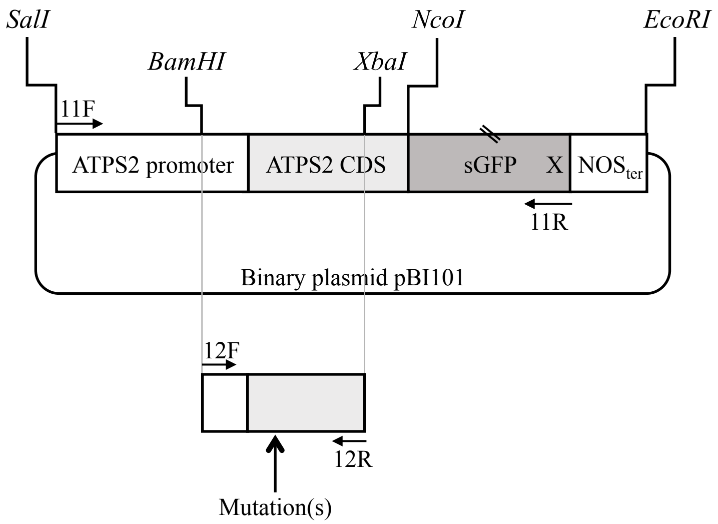


**Figure S1.** Schematic representation of *ATPS2pro:ATPS2-sGFP:NOSter* fusion gene construct. Positions of annealing sites of primers (11F, 11R, 12F and 12R) and a stop codon of GFP (X) are indicated. Sequences of the primers used are detailed in Supplemental Table S4. The combinations of DNA template and primers used for introducing mutations are listed in Supplemental Table S5.
