# Supplementary material for: Alternative translational initiation of ATP sulfurylase underlying dual localization of sulfate assimilation pathways in plastids and cytosol in Arabidopsis thaliana
Source: Front Plant Sci. 2015 Jan 5;5:750. doi: 10.3389/fpls.2014.00750 (PMC4283515; doi:10.3389/fpls.2014.00750)
Supplement: Supplementary file 7 [file Presentation2.PDF]

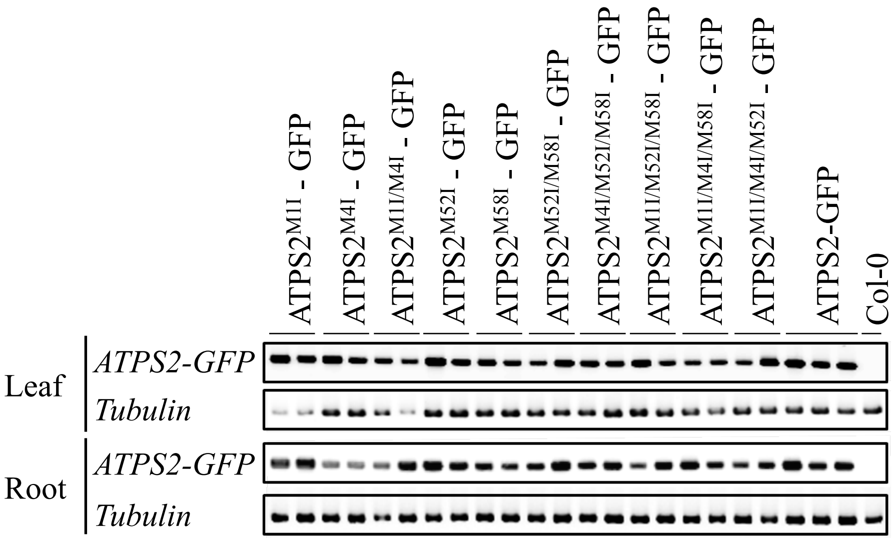

Figure S2. *ATPS2-GFP* mRNA accumulation in leaves and roots of *Arabidopsis* transgenic lines determined by RT-PCR.

First-strand cDNAs were synthesized from total RNAs by reverse transcription using oligo-dT primers, and used as templates for PCR amplification of double-stranded cDNAs derived from the *ATPS2-GFP* and the α-tubulin (TUA3; AT5G19770; Ludwig et al., 1997) gene transcripts. The signals detecting α-tubulin transcripts represent internal standards for normalization of RNA concentrations. The sequences of gene-specific primer pairs are shown below.
ATPS2-F (5’-CTTACGTTTCTCACACTCACTCTCTTC-3’)
GFP-R (5’-TTACTTGTACAGCTCGTCCATGCCGAG-3’)
TUB-F29 (5′-CTCGAAATTAGGGTTTCTACTGAGAGAAG-3′)
TUB-R29 (5′-CCGAACGAATATTTTACAGGATTTAAACA-3′)

Ludwig, S.R., Oppenheimer, D.G., Silflow, C.D., and Snustad, D.P. (1997). Characterization of the alpha-tubulin gene family of *Arabidopsis thaliana*. *Proc. Natl. Acad. Sci. U. S. A.* 84, 5833-7.
